# Comparison of clinical features and pregnancy outcomes in early- and late-onset preeclampsia with HELLP syndrome: a 10-year retrospective study from a tertiary hospital and referral center in China

**DOI:** 10.1186/s12884-022-04466-9

**Published:** 2022-03-08

**Authors:** Boya Li, Huixia Yang

**Affiliations:** Department of Obstetrics and Gynecology, Peking University First Hospital, Beijing, China

**Keywords:** Preeclampsia, Early- and late-onset preeclampsia, HELLP syndrome, Clinical features, Pregnancy outcomes

## Abstract

**Background:**

Early-onset preeclampsia (EO-PE) and late-onset preeclampsia (LO-PE) are different subtypes of preeclampsia. We conducted this study to analyze the similarities and differences in the clinical features and pregnancy outcomes in EO- and LO-PE with HELLP syndrome.

**Methods:**

This was a retrospective study in a tertiary hospital. Eighty-three parturients with HELLP syndrome were allocated into two groups based on the timing of preeclampsia onset: EO-PE with HELLP (*n* = 47) and LO-PE with HELLP (*n* = 36).

**Results:**

In total, 31.9% and 63.9% of women in the EO-PE with HELLP and LO-PE with HELLP groups, respectively, were asymptomatic at diagnosis (*P* = 0.004, OR = 0.265 (0.106–0.662)). Headache or visual symptoms were more frequent in the EO-PE group than in the LO-PE group (48.9% vs. 25%, *P* = 0.026, OR = 0.348 (0.135–0.896)). Women in the EO-PE with HELLP group had higher SBP and DBP than those in the LO-PE with HELLP group. Laboratory tests, including platelets, liver function, and hemolysis, which are the main indicators for the diagnosis of HELLP syndrome, showed almost no significant differences between the two groups, with kidney function being the only difference observed. Women in the EO-PE with HELLP group had higher Scr than those in the LO-PE with HELLP group. The degree of proteinuria was higher in the EO-PE group than in the LO-PE with HELLP group. The incidence of severe maternal complications was significantly higher in the EO-PE group than in the LO-PE with HELLP group (25.5% vs. 5.6%, *P* = 0.016, OR = 0.172 (0.036–0.824)). In total, 57.4% and 8.3% of neonates in the EO-PE and LO-PE with HELLP groups were admitted to the NICU, and the difference was statistically significant, even after adjustment for the delivery week (*P* = 0.009, OR = 0.830 (0.729–0.944)). Postpartum HELLP syndrome was more common in the LO-PE group than in the EO-PE group (30.6% vs. 4.3%, *P* = 0.001, OR = 9.9 (2.031–48.256)).

**Conclusions:**

Compared with LO-PE with HELLP patients, EO-PE with HELLP patients have more obvious kidney damage, higher blood pressure and a higher risk of adverse maternal and neonatal outcomes. Patients with LO-PE need to be alerted to the occurrence of HELLP syndrome after delivery.

**Supplementary Information:**

The online version contains supplementary material available at 10.1186/s12884-022-04466-9.

## Background

Preeclampsia is a heterogeneous syndrome that affects 3–5% of pregnancies [1]. It is usually characterized by hypertension and proteinuria after 20 weeks of gestation and may lead to multisystem disorders. It is one of the main causes of maternal, fetal, and neonatal mortality worldwide [2].

Early-onset preeclampsia (EO-PE) and late-onset preeclampsia (LO-PE) have different characteristics and are generally considered to be subtypes of preeclampsia. Regarding the timing of disease onset, EO-PE occurs before 34 weeks of gestation, and LO-PE occurs at 34 weeks of gestation or later [3]. Genetic association studies suggest that EO-PE is more strongly associated with intrinsic placental factors, while LO-PE is more strongly associated with predisposing maternal factors rather than placental involvement [1], which suggests that the two subtypes may be different forms of preeclampsia with different etiologies. The two subtypes also have different risk factors and may lead to different pregnancy outcomes [4]. However, studies that compare clinical parameters or laboratory biomarkers between EO- and LO-PE are limited [5].

HELLP syndrome is a severe form of preeclampsia characterized by microangiopathic hemolysis, abnormal liver function tests, and thrombocytopenia, with or without proteinuria or severe hypertension [6]. It often has an acute onset, with rapid and sometimes sudden deterioration of the maternal and fetal conditions, and may cause serious complications without early recognition and appropriate clinical intervention. The presence of HELLP syndrome is associated with an increased risk of maternal death and increased rates of maternal morbidities such as acute renal failure, disseminated intravascular coagulopathy (DIC), placental abruption, liver hemorrhage or failure and stroke [7].

Since it was first described by Weinstein in 1982 [8], numerous studies have been published in an attempt to refine the diagnostic criteria for this syndrome, to identify risk factors for adverse pregnancy outcomes and to reduce maternal and perinatal outcomes in women with this syndrome. However, studies investigating the differences in clinical features and pregnancy outcomes of HELLP syndrome between different subtypes of preeclampsia are limited.

Given that EO and LO-PE are different subtypes of preeclampsia, we conducted this study to analyze the similarities and differences in the clinical symptoms, laboratory biomarkers, clinical course, recovery times, and maternal and neonatal outcomes between EO and LO-PE with HELLP syndrome.

## Methods

### Study design and diagnostic criteria for HELLP syndrome

This retrospective study included 86 women with HELLP syndrome admitted to our hospital within the last decade, from April 1^st^, 2009, to March 31^st^, 2019.

The diagnostic criteria used for HELLP syndrome are variable and inconsistent. Traditionally, in China, the criteria for the diagnosis of HELLP syndrome require the presence of the following laboratory findings [9]: (1) hemolysis, defined by abnormal peripheral smear, increased bilirubin (≥ 20.5 μmol/L), slight decreased hemoglobin and increased lactic dehydrogenase. (2) elevated liver enzymes, defined as ALT ≥ 40 U/L or AST ≥ 70 U/L. (3) low platelets, defined as platelet (PLT) count < 100 × 10^9^/L. However, internationally, the Tennessee classification system was widely used to diagnose this syndrome[10], which includes the following laboratory findings: (1) hemolysis, defined by abnormal peripheral smear, increased bilirubin (≥ 20.5 μmol/L), and increased lactic dehydrogenase (LDH ≥ 600 U/L); (2) elevated liver enzymes, defined as increased serum aspartate aminotransferase levels (AST ≥ 70 U/L) and increased LDH; (3) low platelets, defined as PLT˂100 × 10^9^/L.

In this study, we used the Tennessee classification system to diagnose HELLP syndrome. A review of the medical records of the 86 parturients who met the diagnostic criteria of our country resulted in the exclusion of three of them because they did not meet the criteria of the Tennessee classification system and the inclusion of 83 parturients in the analysis. These parturients were then allocated into two groups based on the timing of preeclampsia onset: EO-PE with HELLP syndrome (EO-PE with HELLP group, *n* = 47) and LO-PE with HELLP syndrome (LO-PE with HELLP group, *n* = 36).

### Ethics approval

This study was performed in accordance with the Declaration of Helsinki and was reviewed and approved by the Ethics committee of Peking University First Hospital (Reference Number: 2020 research 434). The requirement for informed consent was waived by the ethics committee due to the retrospective nature of the study.

### Definitions

Preeclampsia occurring at less than 34 weeks of gestation was identified as early-onset disease, whereas preeclampsia that occurred at 34 weeks or later was labeled late-onset disease, irrespective of the gestational week at delivery [3]. Complete HELLP syndrome was defined by the presence of all the three criteria mentioned in the Tennessee classification cystem [10]. Partial HELLP syndrome was defined by the presence of one or two features of HELLP but not the complete syndrome [10]. Severe hypertension refers to systolic blood pressure (SBP) ≥ 160 mmHg or diastolic blood pressure (DBP) ≥ 110 mmHg or both [9]. Acute renal failure (ARF) refers to an elevated serum creatinine (Scr) level of greater than 2 mg/L (176.8 μmol/L) [11]. Thrombocytopenia refers to PLT less than 100 × 10^9^/L [9]. Disseminated intravascular coagulopathy (DIC) was defined by the presence of three or more of the following laboratory findings: Plt ˂100 × 10^9^/L, low fibrinogen (FIB ˂ 3 g/L), positive D-dimer (≥ 400 mg/L), prolonged prothrombin time (PT ≥ 14 s) or prolonged activated partial thromboplastin time (APTT ≥ 40 s)[11]. Severe maternal complications referred to any of the following occurrences: (1) episode(s) of eclampsia, (2) placental abruption, (3) pulmonary edema, (4) heart failure, (5) DIC, (6) ARF, (7) maternal death, or (8) admission to the intensive care unit (ICU). Adverse maternal outcomes refers to the presence of any severe maternal complications or transfusion. Fatal induction of labor refers to termination of pregnancy in the second trimester, in most cases, before 28 gestational weeks. In China, the cutoff time for the perinatal period is 28 gestational weeks.

### Data collection

The collected data included demographic parameters, obstetric history, arterial pressure, clinical symptoms, laboratory biomarkers, clinical course of the disease, route of delivery, recovery time, treatment, maternal and neonatal outcomes. Maternal outcomes collected included episode(s) of eclampsia, placental abruption, pulmonary edema, heart failure, DIC, ARF, maternal death, admission to ICU and transfusion. Transfusion included administration of whole packed erythrocytes, platelets and fresh frozen plasma. The decision to perform transfusion was made on both clinical and hematological parameters. Neonatal outcomes collected included birth weight, Apgar score, fetal growth restriction (FGR), admission to the neonatal intensive care unit (NICU), fatal induction of labor, intrauterine death, neonatal death and fetal nonviability.

### Statistical analysis

Statistical analyses for differences of the two groups were performed using independent sample t-test of variance on continuous variables with normal distribution, Mann–Whitney U test on continuous variables with non-normal distribution, and the Chi-square test on categorical variables. Categorical variables with an incidence ≤ 5 were evaluated with the Fisher’s exact test. A probability of ≤ 0.05 was considered to be statistically significant. Descriptive statistics were shown as mean ± standard deviation (SD) or median [25^th^ percentile-75^th^ percentile] for continuous variables. The results for categorical variables were shown as noun and percentage. Fetal and neonatal outcomes were evaluated after adjustment for gestational age at delivery. Statistical analysis was performed using SPSS version 19.0.

## Results

### Baseline demographic characteristics

Table 1 shows the baseline demographic characteristics and risk factors in each group in our study. Thirty percent of women with HELLP syndrome were aged 35 years or more. However, there was no difference in maternal age or the number of women who were over 35 years old between EO-PE and LP-PE with HELLP syndrome (27.7% versus 33.3%). The twin or multiple birth rate in the LO-PE with HELLP group was higher than that in the EO-PE with HELLP group, but the difference was not statistically significant (19.4% vs. 6.4%, *P* = 0.093). A total of 27.8% of women in the LO-PE with HELLP group had pregnancy complicated by with diabetes (including pregestational diabetes mellitus (PGDM) and gestational diabetes mellitus (GDM)), while only 17.0% in the EO-PE with HELLP group had pregnancy-associated diabetes. However, there was no statistically significant difference (*P* = 0.239). Our hospital is a tertiary hospital that accepts referrals from primary hospitals, and the total referral rate was 45.8% among the 83 patients with HELLP. The referral rate for the EO-PE with HELLP group was significantly higher than that for the LO-PE with HELLP group (61.7% vs. 25.7%, *P* = 0.001, OR = 0.215 (0.082–0.561)). There were no differences in maternal weight or BMI before delivery, gravidity, primiparity, chronic hypertension, history of preeclampsia or history of fetal growth restriction (FGR) between the two groups.

**Table 1:** Comparison of demographic characteristics and risk factors in EO-PE and LO-PE with HELLP syndrome

	**EO-PE with HELLP group (*n* = 47)**	**LO-PE with HELLP group (*n* = 36)**	***P* value**
Maternal age, years	32.23 (5.42)	31.67 (4.86)	0.623
Maternal Age ≥ 35	13 (27.7%)	12 (33.3%)	0.577
Twin or multiple pregnancies	3 (6.4%)	7 (19.4%)	0.093
Maternal weight before delivery, kg	77.38 (14.99)	74.08 (11.14)	0.242
Maternal BMI before delivery, kg/m^2^	28.91 [26.02–32.90]	28.30 [25.89–31.46]	0.416
Gravidity	2 [1-3]	2 [1-3]	0.449
Primiparity	29 (61.7%)	24 (66.7%)	0.641
Chronic hypertension	9 (19.1%)	5 (13.9%)	0.570
Pregnancy associated with diabetes (PGDM or DM)	8 (17.0%)	10 (27.8%)	0.239
History of preeclampsia	5 (10.6%)	3 (8.3%)	0.724
History of FGR	2 (4.3%)	0	0.503
**Referral**	**29 (61.7%)**	**9 (25.7%)**	0.001*

Data are given as mean (SD) or median [25^th^ percentile-75^th^ percentile] or number (percentage). * *P* ˂ 0.05

### Clinical course and recovery time

Table 2 shows the clinical course of preeclampsia and the recovery time in each group. The mean ± SD of the timing for preeclampsia onset in the EO-PE and LO-PE with HELLP groups was 28.66 ± 3.35 and 37.30 ± 2.12 gestational weeks, while the mean ± SD of delivery week was 29.43 ± 3.39 and 37.47 ± 1.89 gestational weeks, respectively. The median [25th percentile–75th percentile] times from preeclampsia onset to delivery and from diagnosis of HELLP syndrome to delivery were 2.97 [0.98–7] and 1.00 [0–2.00] days for the EO-PE with HELLP group, respectively, which were significantly higher than those of the LO-PE with HELLP group (*P* = 0.002). The total cesarean delivery rate was 80.0% for HELLP syndrome in our study. However, there was no difference in the cesarean delivery rate between EO-PE and LO-PE with HELLP syndrome (72.3% versus 88.9%).

**Table 2:** Comparison of clinical course and recovery time in EO-PE and LO-PE with HELLP syndrome

	**EO-PE with HELLP group (*n* = 47)**	**LO-PE with HELLP group (*n* = 36)**	***P* value**
**Onset of PE, wks**	**28.66 (3.35)**	**37.30 (2.12)**	< 0.001*
**Delivery week, wks**	**29.43 (3.39)**	**37.47 (1.89)**	< 0.001*
**From PE onset to delivery, days**	**2.97 [0.98–7]**	**0 [0–2.49]**	0.002*
**From diagnosis of HELLP to delivery (not including postpartum HELLP), days**	**1.00 [0–2.00]**	**0 [0–0]**	< 0.001*
Cesarean delivery	34 (72.3%)	32 (88.9%)	0.064
Discharge time after delivery, days	5 [4-7]	5 [4-7]	0.993
According to complete or partial HELLP syndrome			
Complete HELLP syndrome	16 (34.0%)	7 (19.4%)	0.141
Partial HELLP syndrome	31 (66.0%)	29 (80.6%)	0.141
According to antenatal or postpartum HELLP syndrome			
**Antenatal**	**45 (95.7%)**	**25 (69.4%)**	0.001*
**Postpartum**	**2 (4.3%)**	**11 (30.6%)**	0.001*

Data are given as mean (SD) or median [25^th^ percentile-75^th^ percentile] or number (percentage). * *P* ˂ 0.05

Because the level of platelet or liver function in some patients did not return to normal before discharge, we used discharge time instead of recovery time of organ function to reflect recovery from the disease. There were no differences in discharge time after delivery (5 [4-7] vs. 5 [4-7], *P* = 0.993) between the two groups.

In the EO-PE and LO-PE with HELLP groups, complete and partial HELLP syndromes accounted for 34.0% and 66.0% vs. 19.4% and 80.6% of patients (*P* = 0.141), respectively. Postpartum HELLP syndrome was more common in the LO-PE with HELLP group than in the EO-PE with HELLP group (30.6% vs. 4.3%, *P* = 0.001, OR = 9.9 (2.031–48.256)). In addition, among the 13 cases of postpartum HELLP syndrome, 5 cases were twin pregnancies, accounting for 38% of cases of postpartum HELLP syndrome.

### Clinical symptoms and signs

Table 3 shows the comparison of clinical symptoms and signs between EO-PE and LO-PE with HELLP syndrome. A total of 31.9% of women in the EO-PE with HELLP group and 63.9% in the LO-PE with HELLP group were asymptomatic at diagnosis (*P* = 0.004, OR = 0.265 (0.106–0.662)). Headache or visual symptoms were more frequent in the EO-PE group than in the LO-PE with HELLP group (48.9% vs. 25%, *P* = 0.026, OR = 0.348 (0.135–0.896)). There were no differences in the appearances of clinical symptoms, including upper abdominal pain, palpability or suffocation, nausea or vomiting, or vaginal bleeding, between the EO-PE and LO-PE with HELLP groups.

**Table 3:** Comparison of clinical symptoms and signs in EO-PE and LO-PE with HELLP syndrome

Symptoms and signs	EO-PE with HELLP group (*n* = 47)	LO-PE with HELLP group (*n* = 36)	*P*	Odds ratio (CI)
**Asymptomatic at diagnosis**	**15 (31.9%)**	**23 (63.9%)**	0.004*	**0.265 (0.106–0.662)**
**Headache or visual symptoms**	**23 (48.9%)**	**9 (25%)**	0.026*	**0.348 (0.135–0.896)**
Upper abdominal pain	11 (23.4%)	5 (13.9%)	0.276	
Palpability or suffocation	4 (8.5%)	3 (8.3%)	0.977	
Nausea or vomiting	10 (21.3%)	4 (11.1%)	0.220	
Vaginal bleeding	0	1 (2.8%)	0.434	
**AREDF**	**7 (15.6%)**	**0 (0%)**	0.013*	**0.844 (0.745–0.957)**
**Highest SBP before delivery**	**179.83 (25.12)**	**162.69 (18.65)**	0.001*	
**Highest DBP before delivery**	**108.64 (17.66)**	**99.03 (13.93)**	0.004*	
**Severe hypertension**	**39 (83.0%)**	**19 (52.8%)**	0.003*	**0.229 (0.084–0.625)**

Data are given as number (percentage). * *P*˂0.05

Women in the EO-PE with HELLP group had higher SBP and DBP than those in the LO-PE with HELLP group. Seven (15.6%) women in the EO-PE with HELLP group had absent and/or reversed end-diastolic flow in the umbilical artery (AREDF), while none had it in the LO-PE with HELLP group (*P* = 0.013, OR = 0.844 (0.745–0.957)).

### Laboratory indexes

Laboratory biomarkers of platelets, hemolysis, and liver and renal functions between EO-PE and LO-PE with HELLP syndrome are summarized in Table 4. The average levels of PLT, ALT, AST, Tbil, ALB, LDH and FIB between groups were all similar. When we converted PLT, ALT and LDH into categorical variables, the results were consistent with the former. The only difference was in kidney function. Women in the EO-PE with HELLP group had higher Scr than those in the LO-PE with HELLP group (84.19 ± 34.37 vs. 70.24 ± 19.30 mol/l, *P* = 0.032). Both qualitative and quantitative detection of urinary protein showed that the degree of proteinuria was higher in the EO-PE group than in the LO-PE with HELLP group.

**Table 4:** Comparison of laboratory indexes in EO-PE and LO-PE with HELLP syndrome

	**EO-PE with HELLP group (*n* = 47)**	**LO-PE with HELLP group (*n* = 36)**	***P***
ALT, IU/L	91 [41–236]	58 [12–170]	0.131
AST, IU/L	91 [41–218]	64 [23–198]	0.131
Tbil, μmol/L	9.4 [5.5–21.33]	11.3 [6.3–21.2]	0.620
ALB, g/L	28.36 (3.99)	29.05 (3.63)	0.424
LDH, U/L	403.50 [298.25–717.75]	405.0 [232.25–709.75]	0.270
PLT, 10^9^/L	81 [59–92]	81.5 [62–94]	0.538
**Scr,** µmol/L	**84.19 (34.37)**	**70.24 (19.30)**	0.032*
Fib, mg/L	3.46 (0.82)	3.63 (0.88)	0.377
**Urine protein, +**	**3 [2–4]**	**2 [1–3]**	0.003*
**24-h proteinuria**	**6.01 (5.49)**	**1.65 (1.37)**	< 0.001*
**Proteinuria ≥ 2 g/24 h**	**25 (71.4%)**	**8 (36.4%)**	0.009*
Plt ˂ 100 × 10^9^/L	41 (87.2%)	30 (83.3%)	0.617
ALT > 70 IU/L	29 (61.7%)	17 (47.2%)	0.188
Scr > 97.25 µmol/L	10 (21.3%)	4 (11.1%)	0.220
LDH > 600 U/L	16 (34.8%)	11 (30.6%)	0.686

Data are given as mean (SD) or median [25^th^ percentile-75^th^ percentile] or number (percentage). * *P* ˂ 0.05

### Adverse maternal outcomes

As shown in Table 5, we compared different maternal levels between EO-PE and LO-PE with HELLP syndrome. Although the incidences of maternal death, episodes of eclampsia, placental abruption, pulmonary edema, heart failure, DIC and ARF were similar between the two groups (Table 5), we found that there were no women with pregnancies complicated by maternal death, placental abruption, heart failure, ARF or DIC in the LO-PE with HELLP group. In other words, the only severe maternal complication in the LO-PE group was eclampsia. A total of 17.0% of women in the EO-PE with HELLP group were admitted to the ICU, while no such admissions occurred in the LO-PE with HELLP group (*P* = 0.009, OR = 0.830 (0.729–0.944)). The incidence of severe maternal complications was significantly higher in the EO-PE with HELLP group than in the LO-PE with HELLP group (25.5% vs. 5.6%, *P* = 0.016, OR = 0.172 (0.036–0.824)).

**Table 5:** Comparison of maternal outcomes in EO-PE and LO-PE with HELLP syndrome

Maternal complications N (%)	EO-PE with HELLP group (*n* = 47)	LO-PE with HELLP group (*n* = 36)	*P*	Odds ratio (CI)
Maternal death	0	0	-	
Episode of eclampsia	3 (6.4%)	2 (5.6%)	1.000	
Placental abruption	2 (4.3%)	0	0.503	
Pulmonary edema	0	0	-	
Heart failure	2 (4.3%)	0	0.503	
ARF	2 (4.3%)	0	0.503	
DIC	2 (4.3%)	0	0.503	
**Admission to the ICU**	**8 (17.0%)**	**0**	0.009*	**0.830 (0.729–0.944)**
**Severe maternal complications**	**12 (25.5%)**	**2 (5.6%)**	0.016*	**0.172 (0.036–0.824)**
**Transfusion**	**35 (74.5%)**	**14 (38.9%)**	0.001*	**0.218 (0.085–0.557)**
**Adverse maternal outcomes**	**36 (76.6%)**	**15 (41.7%)**	0.001*	**0.218 (0.085–0.562)**

Data were given as number (percentage). * *P* < 0.05

The risk of blood transfusion in the EO-PE with HELLP group was almost double that in the LO-PE group (74.5% vs. 38.9%, *P* = 0.001). Furthermore, the incidence of adverse maternal outcomes was significantly higher in the EO-PE group than in the LO-PE with HELLP group (76.6% vs. 41.7%, *P* = 0.001, OR = 0.218 (0.085–0.562)).

### Neonatal outcomes

The neonatal outcomes in EO-PE and LO-PE with HELLP syndrome are summarized in Table 6. The average neonatal birth weights in the EO-PE and LO-PE with HELLP groups were 1142 ± 480 g and 2743 ± 567 g, respectively. The Apgar scores at both 1 min and 5 min were significantly higher in the LO-PE group than in the EO-PE with HELLP group. A total of 57.4% of infants in the EO-PE with HELLP group were admitted to the NICU, while only 8.3% in the LO-PE with HELLP group were admitted; this difference was significant, even after adjustment for delivery week (*P* = 0.009, OR = 0.830 (0.729–0.944)). Although the risk of FGR in the EO-PE with HELLP group was almost double that in the LO-PE with HELLP group, the difference was not statistically significant (28.3% vs. 13.9%, *P* = 0.161).

**Table 6:** Comparison of neonatal outcomes in EO-PE and LO-PE with HELLP syndrome

	**EO-PE with HELLP group (*n* = 47)**	**LO-PE with HELLP group (*n* = 36)**	***P***	**Odds ratio (CI)**	**Adjusted *P***
**Birth weight, g**	**1142 (480)**	**2743 (567)**	< 0.001*		
**1 min Apgar**	**5.50 [0–9.00]**	**10 **[10–10]	< 0.001*		
**5 min Apgar**	**9 [0–10]**	**10 **[10–10]	< 0.001*		
FGR	13 (28.3%)	5 (13.9%)	0.197		0.960
**Admission to the NICU**	**27 (57.4%)**	**3 (8.3%)**	< 0.001*	**0.067 (0.018–0.251)**	< 0.001*
**fatal induction of labor**	**12 (25.5%)**	**0**	0.001*	**0.745 (0.630–0.880)**	**0.998**
intrauterine death,	2 (4.3%)	0	0.503		0.998
neonatal death	4 (9.3%)	0	0.060		0.998
Fetal nonviability	**18 (41.9%)**	**0**	< 0.001*	**0.510 (0.303–0.554)**	**0.998**

Data are given as mean (SD) or median [25^th^ percentile-75^th^ percentile] or number (percentage). The adjusted *P* values were data adjusted for delivery week. * *P* ˂ 0.05

We found that there were no women with pregnancies complicated by fatal induction of labor, intrauterine death or neonatal death in the LO-PE with HELLP group. The incidences of fatal induction of labor, intrauterine death, neonatal death and fetal nonviability were significantly higher in the EO-PE with HELLP group than in the LO-PE with HELLP group. However, after adjustment for delivery week, these variables were all similar between the two groups.

## Discussion

### Risk factors

From the perspective of etiology, EO-PE and LO-PE share some common characteristics but have different etiological features and risk factors, leading to different outcomes. EO-PE is more closely linked to placental factors, while LO-PE is more correlated with maternal factors. A large population-based epidemiologic study showed that several factors, including diabetes mellitus, nulliparity, and young maternal age, were associated with a higher risk of LO-PE, whereas chronic hypertension conferred a higher risk of EO-PE [4]. In our study, in the LO-PE with HELLP group, the incidence of pregnancy complicated by diabetes was 1.5 times that in the EO-PE with HELLP group, and in the EO-PE with HELLP group, the incidence of pregnancy complicated by chronic hypertension was 1.4 times that in the EO-PE with HELLP group, although neither difference was statistically significant, which may be due to the limited sample size. There were no differences in nulliparity or young maternal age between the two groups. Interestingly, we found that nearly one-third of women with HELLP syndrome in our study were aged 35 years or more, regardless of the timing of preeclampsia onset. Maternal age is a risk factor for developing preeclampsia [12]. Our data suggested that advanced maternal age may be associated with HELLP syndrome but may not be associated with the time of preeclampsia onset with HELLP syndrome.

A population-based cohort study revealed that multiple pregnancy was associated with HELLP syndrome in both the first and second pregnancies [13]. In our study, 12% of women with HELLP syndrome had a twin or multiple pregnancy. Although there was no significant difference, the twin or multiple birth rate in the LO-PE with HELLP group was three times that in the EO-PE group.

### Clinical course

In terms of disease course, almost all women in the LO-PE with HELLP group gave birth on the day of diagnosis of HELLP syndrome, while most women in the EO-PE with HELLP group gave birth during or after the administration of corticosteroids to accelerate fetal lung maturity. According to our hospital’s protocol, for women with pregnancies complicated by HELLP syndrome at less than 34 gestational weeks, when the condition is stable and lung maturation treatment has not been performed before or has been completed for more than 2 weeks, 48 h of corticosteroid treatment can be expected to promote fetal lung maturity under close monitoring. During anticipatory treatment, changes in laboratory biomarkers should be dynamically monitored every 6–12 h. For women with pregnancies complicated by HELLP syndrome at more than 34 gestational weeks, prompt delivery should be considered. Thus, in our study, the average number of days from diagnosis of HELLP syndrome to delivery in the EO-PE with HELLP group was significantly higher than that in the LO-PE with HELLP group.

Regarding the mode of delivery, the cesarean section rate of the LO-PE with HELLP group was slightly higher than that of the EO-PE group, although this difference was not statistically significant. In China, 28 gestational weeks is the standard for entering the perinatal period. Most women with pregnancies complicated by HELLP syndrome at less than 28 weeks might choose lethal induction to terminate their pregnancy instead of cesarean section, considering the higher morbidity and mortality among extremely premature infants.

Regarding disease recovery, there was no difference in the discharge times of the two groups, with an average of approximately 5 days in our study, meaning that after delivery of the fetus and placenta, the recovery rate from the disease was similar regardless of the timing of disease onset.

HELLP syndrome is divided into two subtypes according to the Tennessee standards: complete type and partial type. In our study, the proportions of subtypes were not significantly different between the EO-PE and LO-PE with HELLP groups, which reveals that the subtype of HELLP syndrome may not relate to the time of preeclampsia onset.

According to previous reports, 70% of HELLP syndromes occur antepartum and 30% postpartum [14]. In our study, postpartum HELLP syndrome accounted for 30.6% in the LO-PE with HELLP group and only 4.3% in the EO-PE with HELLP group. Therefore, according to our research, postpartum HELLP is more likely to occur in LO-PE. The probability of postpartum HELLP in EO-PE is extremely low. This may be because for EO-PE without HELLP syndrome, expectant treatment before 34 weeks was permitted when the condition was stable. For severe preeclampsia after 34 weeks, delivery is the optimal choice. Our research suggests that for severe preeclampsia after 34 weeks, the possibility of HELLP syndrome still needs to be monitored for 1–4 days, especially during the first 24 h after delivery.

### Clinical symptoms and laboratory tests

Compared with the LO-PE with HELLP group, the EO-PE group more often had clinical symptoms at diagnosis, accounting for approximately two-thirds of patients. Typical clinical symptoms of HELLP syndrome are pain in the right upper quadrant of the abdomen or epigastric pain, nausea and vomiting [15]. Patients frequently have right upper quadrant or epigastric pain, nausea, or vomiting ranging in frequency from 30 to 90%, headaches are reported by 33–61% [7] of patients, and visual changes are reported in approximately 17%. In our research, headache or visual symptoms were the most common symptoms in both groups. The risk of headache or visual symptoms in the EO-PE with HELLP group was twice that of the LO-PE with HELLP group. The reason for this may be that the former patients are more vulnerable to severe hypertension. In addition, the risks of upper abdominal pain and nausea or vomiting in the LO-PE with HELLP group were higher than those in the EO-PE with HELLP group, but the difference was not statistically significant.

Umbilical artery blood flow could reflect placental function to a certain extent. In our study, the risk of AREDF in the EO-PE with HELLP group was significantly higher than that in the LO-PE with HELLP group, which reflects a feature of EO-PE in that it is more closely associated with placental factors.

Laboratory tests, including platelets, liver function, and hemolysis, are the main indicators for the diagnosis of HELLP syndrome. However, our research showed that there was no significant difference in these biomarkers between the two groups, which means that there is no significant correlation between the time of preeclampsia onset and the severity of HELLP. A previous study showed that renal function was significantly higher EO-PE [5]. Consistent with that, our research showed that Scr and the severity of proteinuria were significantly higher in the EO-PE with HELLP group than in the LO-PE with HELLP group. A previous study showed that the pathomechanism of endothelial damage seemed to be more sFlt-1 dependent in patients with EO-PE than in patients with LO-PE [16]. The severity of proteinuria reflected the degree of kidney glomerular endothelial cell damage. The higher severity of proteinuria in the EO-PE with HELLP group is probably a consequence of more sFlt-1-dependent endothelial damage in the EO-PE with HELLP group than in the LO-PE with HELLP group. Proteinuria seems to be the best renal predictive factor of PE [17]. It may predict the severity of preeclampsia in clinical practice.

### Pregnancy outcomes

The presence of HELLP syndrome is notoriously associated with both maternal and neonatal adverse outcomes, with the only effective treatment being prompt delivery of the baby.

In our study, the incidence of adverse maternal outcomes for HELLP syndrome was 61.4%. The risk of adverse maternal outcomes in the EO-PE with HELLP group was 1.7 times that in the LO-PE group. The most common adverse maternal outcome was transfusion in both groups, as reported previously by others [18, 19]. A high rate of transfusion may be associated with thrombocytopenia, placental abruption, postpartum hemorrhage, DIC, correct hypoalbuminemia and the need for plasma exchange.

The total incidence of severe maternal complications with HELLP syndrome was 16.8%. In addition to entering the ICU for various reasons, eclampsia was the most common severe maternal complication, with an incidence of 6.0%. We were surprised to find that the only two patients with severe maternal complications in the LO-PE with HELLP group had eclampsia. There were no women with pregnancies complicated by maternal death, placental abruption, heart failure, pulmonary edema, DIC or ARF in the LO-PE with HELLP group. Even in the EO-PE with HELLP group, the incidences of maternal death, eclampsia, placental abruption, DIC and ARF in our hospital were significantly lower than those in other literature reports [7, 16]. This may be due to our comprehensive understanding of HELLP, rich experience, timely delivery and multidisciplinary cooperation as a tertiary hospital and referral center for high-risk pregnant women.

Preeclampsia, especially EO-PE, is usually associated with insufficient placentation, which may result in FGR. In our study, the risk of FGR in the EO-PE with HELLP group was twice as high as that in the LO-PE group, although the difference was not statistically significant. However, the incidence of FGR in our study was lower than that in other studies [4, 20]. Since our study included women with HELLP syndrome from April 1st, 2009, to March 31st, FGR was defined as birth weight below the 10th percentile in accordance with our hospital guidelines. In June 2019, the Fetal Medicine Subgroup, Society of Perinatal Medicine, Chinese Medical Association and Obstetrics Subgroup, Society of Obstetrics and Gynecology, Chinese Medical Association released expert consensus on fetal growth restriction and recommended using the NICHD fetal growth standard (Asian) to diagnose FGR [21, 22]. We then compared our hospital standard with the NICHD standard (Asian) in Table S1 and found that using our hospital old standard would underestimate the incidence of FGR (Table S2). When we used the NICHD standard (Asian), the risk of FGR in the EO-PE with HELLP group was almost three times that of the LO-PE with HELLP group (66.0% vs. 22.2%), and it showed statistical significance even after adjustment for delivery week (*P* = 0.013, OR = 0.121 (0.023–0.643)).

The incidences of fatal induction of labor, intrauterine death, neonatal death and fetal nonviability were all similar between the two groups after adjustment for delivery week. This may be due to the greater correlation between gestational age at delivery and neonatal outcome compared with the disease itself. It is worth noting that even after adjusting for the gestational week of delivery, the risk of neonates entering the NICU in the EO-PE with HELLP group was still significantly higher than that in the LO-PE with HELLP group, which means that the EO-PE with HELLP group had a higher perinatal morbidity than the LO-PE with HELLP group.

## Conclusions

Compared with patients with LO-PE with HELLP syndrome, patients with EO-PE with HELLP syndrome have more obvious kidney damage and higher blood pressure, are more likely to have clinical symptoms, especially headache and visual symptoms, often have concomitant AREDF, and have a higher risk of adverse maternal and neonatal outcomes. Patients with LO-PE need to be alerted to the occurrence of HELLP syndrome after delivery.

## Supplementary Information

**Additional file 1: Table S1**. Comparison of the 10th percentile between our hospital and the NICHD standard (Asian). **Table S2.** Comparison of different standard to diagnose FGR.

## Data Availability

The datasets analyzed during the current study are available from the corresponding author on reasonable request.

## Abbreviations

EO-PE: Early-onset preeclampsia
LO-PE: Late-onset preeclampsia
HELLP: Hemolysis, elevated liver enzymes and low platelets
DIC: Disseminated intravascular coagulopathy
SBP: Systolic blood pressure
DBP: Diastolic blood pressure
ARF: Acute renal failure
Scr: Serum creatinine
FIB: Fibrinogen
PT: Prolonged prothrombin time
APTT: Activated partial thromboplastin time
ICU: Intensive care unit
PLT: Platelet
LDH: Lactic dehydrogenase
FGR: Fetal growth restriction
NICU: Neonatal intensive care unit
SD: Standard deviation
AREDF: Absent and/or reversed end-diastolic flow in the umbilical artery
