# Supplementary material for: Comparison of clinical features and pregnancy outcomes in early- and late-onset preeclampsia with HELLP syndrome: a 10-year retrospective study from a tertiary hospital and referral center in China
Source: BMC Pregnancy Childbirth. 2022 Mar 8;22:186. doi: 10.1186/s12884-022-04466-9 (PMC8903662; doi:10.1186/s12884-022-04466-9)
Supplement: Supplementary file 1 — Additional file 1: Table S1. Comparison of the 10th percentile between our hospital and the NICHD standard (Asian). Table S2. Comparison of different standard to diagnose FGR. [file 12884_2022_4466_MOESM1_ESM.docx]

**Supplementary Information**

Table S1 Comparison of the 10th percentile between our hospital and the NICHD standard (Asian)

| Gestational weeks | Our hospital  10th percentile (g) | NICHD(Asian)  10th percentile (g) |
| --- | --- | --- |
| 22 | 320 | 394 |
| 23 | 365 | 466 |
| 24 | 417 | 546 |
| 25 | 477 | 637 |
| 26 | 546 | 740 |
| 27 | 627 | 853 |
| 28 | 720 | 978 |
| 29 | 829 | 1114 |
| 30 | 955 | 1260 |
| 31 | 1100 | 1414 |
| 32 | 1284 | 1574 |
| 33 | 1499 | 1740 |
| 34 | 1728 | 1911 |
| 35 | 1974 | 2085 |
| 36 | 2224 | 2262 |
| 37 | 2455 | 2437 |
| 38 | 2642 | 2604 |
| 39 | 2790 | 2752 |
| 40 | 2891 | 2873 |

| FGR | EO-PE with HELLP group (n=47) | LO-PE with HELLP group (n=36) | P | Odds ratio (CI) | Adjusted P |
| --- | --- | --- | --- | --- | --- |
| Our hospital standard | 13 (28.3%) | 5 (13.9%) | 0.197 |  | 0.960 |
| NICHD standard (Asian) | 31（66.0%） | 8（22.2%） | ＜ 0.001* | 0.121（0.023-0.643） | 0.013* |

Table S2 Comparison of different standard to diagnose FGR
